# Partner intrinsic characteristics influence foraging trip duration, but not coordination of care in wandering albatrosses *Diomedea exulans*

**DOI:** 10.1002/ece3.9621

**Published:** 2022-12-15

**Authors:** Fionnuala R. McCully, Henri Weimerskirch, Stephen J. Cornell, Ben J. Hatchwell, Milena Cairo, Samantha C. Patrick

**Affiliations:** ^1^ School of Environmental Sciences University of Liverpool Liverpool UK; ^2^ Centre d'Etudes Biologiques de Chizé Centre National de la Recherche Scientifique Villiers en Bois France; ^3^ Institute of Infection, Veterinary and Ecological Sciences University of Liverpool Liverpool UK; ^4^ School of Biosciences University of Sheffield Sheffield UK; ^5^ Centre d'Ecologie et des Sciences de la Conservation (UMR 7204) Muséum National d'Histoire Naturelle Paris France

**Keywords:** albatrosses, behavioral coordination, foraging bout, nest attendance, parental care, personality, seabirds

## Abstract

Long‐lived monogamous species gain long‐term fitness benefits by equalizing effort during biparental care. For example, many seabird species coordinate care by matching foraging trip durations within pairs. Age affects coordination in some seabird species; however, the impact of other intrinsic traits, including personality, on potential intraspecific variation in coordination strength is less well understood. The impacts of pair members' intrinsic traits on trip duration and coordination strength were investigated using data from saltwater immersion loggers deployed on 71 pairs of wandering albatrosses *Diomedea exulans*. These were modeled against pair members' age, boldness, and their partner's previous trip duration. At the population level, the birds exhibited some coordination of parental care that was of equal strength during incubation and chick‐brooding. However, there was low variation in coordination between pairs and coordination strength was unaffected by the birds' boldness or age in either breeding stage. Surprisingly, during incubation, foraging trip duration was mainly driven by partner traits, as birds which were paired to older and bolder partners took shorter trips. During chick‐brooding, shorter foraging trips were associated with greater boldness in focal birds and their partners, but age had no effect. These results suggest that an individual's assessment of their partner's capacity or willingness to provide care may be a major driver of trip duration, thereby highlighting the importance of accounting for pair behavior when studying parental care strategies.

## Graphical abstract

This investigation into whether individual and partner intrinsic traits influenced parental care coordination in wandering albatrosses (*Diomedea exulans*) found that coordination was equally strong in both incubation and chick‐brooding, but was unaffected by intrinsic traits throughout breeding. Surprisingly, incubation trips were influenced by partners' traits, in that birds with older and bolder partners took shorter trips, while in brooding, short trips were associated with increased boldness of focal birds and partners. This demonstrates the importance of modeling pair behavior when investigating the drivers of trip duration.

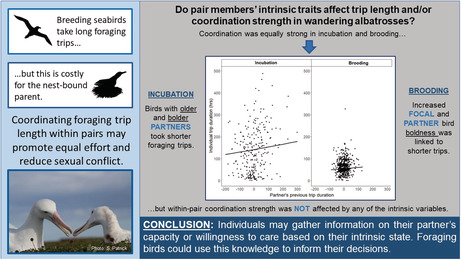

## 1 INTRODUCTION

Avian biparental care is a delicate balancing act, as its high costs are borne by individuals, but the benefits are shared, leading to sexual conflict (Johnstone & Savage, 2019). Much attention has been paid to systems where parents benefit by minimizing their own contribution to care‐giving and allowing their partner to incur the majority of the costs (Houston & Davies, 1985; Trivers, 1972). This strategy is most effective when pair bonds are short‐lived and sacrificing the long‐term condition of a current partner is less likely to accrue fitness costs during future breeding attempts (Barta et al., 2002; Khwaja et al., 2017). In contrast, less attention has been paid to the cooperative aspects of parental care displayed by long‐lived, monogamous species which are particularly vulnerable to the costs of sexual conflict (Griffith, 2019). When environmental conditions and life history increase the cost of changing partners, pair bonds should be maintained between breeding seasons (Bried et al., 2003; Mercier et al., 2021). If parental effort within such species is uneven, one parent's condition may decline, damaging their ability to invest in current and future broods (Griffith, 2019; Royle et al., 2002). This will ultimately affect the pair's lifetime success, as each individual's long‐term fitness is inextricably linked to that of their partner. Behaviors that facilitate equal effort in parental care should therefore be adaptive in species that form long‐lasting partnerships, because they reduce the possibility of one partner incurring heavy costs in comparison to the other (Mariette & Griffith, 2015). Despite this, interindividual differences in behavior often shape decision‐making (e.g., Krüger et al., 2019; Mutzel et al., 2013), and the presence of multiple behavioral phenotypes may prevent the uniform expression of parental care behaviors within a population. Such variation might affect each individual's parental behavior, as well as influencing how pair members respond to one another, thereby creating interpair variation in parental care strategy (Both et al., 2005; Schuett et al., 2011).

Animal personality, characterized by repeatable, individual differences in behavior which are consistent over time (Réale et al., 2007), has been connected to parental care through its impact on provisioning (Mutzel et al., 2013) and mate choice (Schuett et al., 2011). Personality may explain individual variation in foraging strategies in animals (reviewed in Toscano et al., 2016) because bold individuals are thought to be more risk‐tolerant (Dammhahn & Almeling, 2012; Van Oers et al., 2004) and engage in more exploration compared to shy conspecifics (Carter et al., 2013; Verbeek et al., 1994). However, despite the potential for personality to influence parental care, questions remain on how such variation in foraging strategy might impact an individual's participation in cooperative behaviors designed to support equal effort and to protect a partnership's long‐term fitness.

Coordinated parental care has been cited as an example of a behavior that promotes equal effort within pairs (Johnstone et al., 2014; Wojczulanis‐Jakubas et al., 2018). In some passerines which leave young nestlings unattended, coordinated care manifests as synchrony and/or alternation of nest visits (Bebbington & Hatchwell, 2015; Boucaud et al., 2017). In other taxa including seabirds, where one parent is always present at the nest for much of the rearing period, coordination involves taking alternating foraging trips of similar lengths within pairs (Shoji et al., 2015; Tyson et al., 2017). Seabirds often engage in long foraging trips and typically, while one partner is foraging, the other remains on the nest to incubate or brood the chick (Takahashi et al., 2017; Weimerskirch et al., 2000). The land‐bound partner loses mass over time, which may eventually cause desertion (Jones et al., 2002; Weimerskirch et al., 1994) or harm that individual's ability to contribute to future broods (Tyson et al., 2017). By matching trip durations, parents should spend an approximately equal time on the nest, causing pair members to incur similar costs (Kavelaars et al., 2019). The potential mechanisms underpinning coordination are varied, as previous findings suggest that, while it sometimes stems from active communication between partners (Boucaud et al., 2017; Takahashi et al., 2017), it can also emerge passively based on the pair members' foraging decisions (Gillies et al., 2021; Savage et al., 2017). Parental care coordination could be particularly beneficial to seabirds because they are often long‐lived, monogamous, and subject to high costs when changing partners (Jouventin et al., 1999; Mercier et al., 2021). They therefore stand to gain from behaviors that divide the costs of parental care more evenly, simultaneously protecting the long‐term fitness of both partners and mitigating the risk of partner desertion (Gillies et al., 2021; Wojczulanis‐Jakubas et al., 2018).

Despite its proposed benefits, considerable variation in coordination strength (how closely trip durations or nest attendance patterns are matched within pairs) has been detected within and across seabird populations, which has led researchers to search for the mechanisms driving these discrepancies (e.g., Grissot et al., 2019; Kavelaars et al., 2019; Patrick et al., 2020). As individually repeatable foraging patterns are common in seabirds (Ceia & Ramos, 2015; Phillips et al., 2017), a trade‐off may emerge as parents attempt to balance a highly specialized individual foraging strategy against strengthening coordination. In addition, the foraging bird controls its trip duration and therefore the nest shift duration of its partner (Cornioley et al., 2016). Thus, the foraging bird's decisions influence the parental behavior of the land‐bound parent and the costs they incur (Gillies et al., 2021). Although a pair's precise location on the continuum between strong and weak coordination is determined by its members' foraging decisions, the variables influencing these decisions are poorly understood.

A number of intrinsic traits are thought to affect individual foraging behavior in seabirds (Phillips et al., 2017), including personality (e.g., Harris et al., 2019; Jeffries et al., 2021; Krüger et al., 2019; Patrick & Weimerskirch, 2014). In some seabird species, bold individuals are often more exploratory (e.g., Patrick et al., 2017; Traisnel & Pichegru, 2019); however, it is not known if dedicating additional time to exploration conflicts with the requirements of coordinated care. Addressing this question could have implications for the study of all animals which share care, as it would provide insight into the reproductive priorities of different personality phenotypes through close examination of their decision‐making. For example, although bold individuals may incur immediate fitness gains by weakening coordination in exchange for greater foraging flexibility (Biro & Stamps, 2008; Réale et al., 2010), this additional exploration may come at the expense of the land‐bound partner's condition and the pair's long‐term fitness. Thus, to each individual, the benefits of coordinated care may vary depending on the constraints imposed by their own intrinsic traits.

Additional intrinsic variables are also known to drive foraging strategy in long‐lived seabirds. Age influences seabird foraging behavior through a combination of experience (Daunt et al., 2007; Frankish et al., 2020) and senescence (Lecomte et al., 2010). Age may also be linked to mate familiarity (Bried et al., 2003), and it has been suggested that newly established pairs may cooperate less effectively (Black, 1996). Previously, it was expected that coordination should increase with experience (Brooke, 1978; Fowler, 1995). However, Patrick et al. (2020) reported that less experienced black‐browed albatross *Thalassarche melanophris* were highly coordinated, but that coordination declined with age, possibly because the future fitness interests of the pair became more likely to diverge with an increased probability of partner death. Consequently, it is important to consider the effects of age and mate familiarity when addressing questions concerning the effects of intraspecific variation in foraging strategy and coordination strength in seabirds. Furthermore, if the adaptive value of coordination in seabirds is to prevent desertion (Weimerskirch, 1995), the land‐bound parent's intrinsic traits (e.g., personality and/or age) may also influence the foraging partner's foraging decisions; however, these potential reciprocal influences of one parent on their partner's behavior have yet to be considered.

The wandering albatross *Diomedea exulans* (henceforth “albatross”) provides an ideal study system to investigate questions on the effect of intrinsic variables on foraging trip duration and parental care coordination. Mated pairs have obligate biparental care, a long lifespan and low re‐pairing rate (Jouventin et al., 1999; Sun et al., 2022). Albatrosses also regulate individual trip duration to prevent a critical loss of mass in a fasting partner (Weimerskirch, 1995) and exhibit repeatable personality traits (Patrick et al., 2013) that impact foraging behavior (Patrick & Weimerskirch, 2015). Our study aims to establish whether individual differences in personality and age of both the focal bird and their partner affect individual foraging trip duration. The prevalence of coordinated parental care in albatrosses has yet to be investigated and so we aim to determine whether coordination occurs in our study population and whether coordination strength varies between pairs. We then investigate whether the intrinsic traits of individuals or their partners affect the strength of coordination exhibited by pairs. We predict that an individual's foraging trip durations will be driven by their own intrinsic traits. Bolder birds will be less coordinated with their partners than shyer birds because they will be more willing to risk weakening coordination in exchange for foraging opportunities. We also anticipate that coordination will decline with age, as the benefits of coordination become less profitable and the probability of re‐pairing increases.

## 2 MATERIALS AND METHODS

### 2.1 Study population

The data were collected from the highly philopatric breeding population of albatrosses on Possession Island, in the Crozet archipelago (46.8°S, 51.8°E), which has been monitored since 1966 (Weimerskirch & Jouventin, 1987). Age, sex (determined via sexual dimorphism), and complete partnership histories (i.e., identifying new and established pairs) were known for all individuals. Adults breed biennially in socially monogamous pairs. Parents return to land in November, before laying a single egg in late December or early January (Fressanges du Bost & Segonzac, 1976; Weimerskirch, 1995). An ~78 day incubation period follows before the egg hatches from mid‐March and chick‐brooding commences (duration = ~30 days) (Weimerskirch, 1995). Parents alternate on the nest to incubate the egg or brood the chick until April, when the chick is left alone and fed regularly by the parents (Weimerskirch et al., 2000). During incubation, trips last between 2 and 30 days, during which time adults may travel 3500 km from the colony in search of food (Weimerskirch et al., 2014). In contrast, trips during brooding tend to be shorter (2–4 days) and more local (average max range 256 km) (Weimerskirch et al., 1993) in order to meet the demands of central place foraging. In both breeding stages, parents mainly prey on squid which they obtain at the surface (Weimerskirch et al., 2005). Fledging occurs in November. Adults delay breeding until a minimum of 7 years of age (Weimerskirch, 1992), and may live upward of 50 years.

### 2.2 Data collection

#### 2.2.1 Saltwater immersion logger specifications and attachment

Between 2008 and 2014, saltwater immersion loggers (British Antarctic Survey, Cambridge; 1998–2013) were fitted to the tarsus of one member of 71 breeding pairs, covering 95 breeding attempts (2008: *n* = 15, 2009: *n* = 24, 2010: *n* = 11, 2011: *n* = 16, 2012: *n* = 13, 2013: *n* = 11, 2014: *n* = 5). Fifty pairs were monitored during a single breeding season, while 21 pairs were monitored for multiple breeding seasons. In three cases, both pair members were tagged (2010: *n* = 1, 2011: *n* = 1, 2013: *n* = 1) and the bird which was tagged second was excluded. The loggers distinguished between “wet” periods (the leg and logger are in saltwater) when the bird is sitting on the water, and “dry” periods (the leg and logger are not in saltwater), which represented either flight or presence on land. Two types of loggers were deployed. The first type recorded the proportion of time the logger was underwater throughout every 10‐min period which were then classified as wet (>45 s wet in 10 min) or dry. The second logger type recorded the specific latency between state changes (wet to dry and vice versa). The loggers weighed 0.03% of the average adult male's mass (Weimerskirch et al., 2014). Chick survival rates are extremely high in this species (Weimerskirch et al., 1997) and in this sample, only four of these breeding attempts resulted in failure.

#### 2.2.2 Intrinsic variables

Boldness was measured repeatedly over the previous 10 years by presenting incubating birds with an approaching human (from a 5 m distance) and quantified using an ordinal scale which categorized the birds' behavioral responses from 0 to 5 (0 = no response; 1 = raises head; 2 = rises onto tarsus; 3 = vocalizes 4 = stands up; 5 = vacates nest). Higher scores were associated with bolder birds. This scale has been verified for inclusion in multiple previous papers (Patrick et al., 2013, 2017; Patrick & Weimerskirch, 2015), and through comparison with the results of novel object testing conducted in the same population (S. Patrick, unpublished data). By fitting the fixed effects of observation number, observer identity, and bird ID using a generalized linear model, individual parameter estimates were produced which were then mean‐centered at the population level (see Patrick et al., 2013 for further description). This created a boldness score for each individual. As in previous work (Patrick et al., 2013), we found that females tended to be bolder than males (female mean = 1.93 ± 1.5 SD, male mean = 1.41 ± 1.07); however, there is substantial overlap in the population and so both sexes are represented across the boldness spectrum. As nest attendance patterns in new partnerships may vary when compared with established pairs (Weimerskirch, 1992), this information was also included in these analyses.

Age ranged between seven and 42 years old (mean = 21.14 ± 7.67 SD). As a quadratic relationship between age and coordination strength has been reported in a similar species (Patrick et al., 2020), we included a quadratic representation of age in our analyses. The oldest and youngest age groups were collapsed until there were at least five individuals in the categories at the extremes of the age distribution. This helps to prevent the detection of specious quadratic relationships (Froy et al., 2013). The age categories were collapsed separately for the incubation and brooding data subsets (see Section 2.3.2). The incubation subset's minimum age was 12 (*n* = 5) and the maximum age was 36 (*n* = 6). The brooding subset's minimum age was eight (*n* = 5) while the maximum age was 34 (*n* = 6).

### 2.3 Data management

#### 2.3.1 Quantifying parental care behavior

Based on data from non‐breeding birds, the maximum continuous flight time of this species is estimated at 12 h (H. Weimerskirch, unpublished data). Dry periods longer than this (mean = 85.23 h ± 65.02 SD) indicated that the bird was on land, allowing differentiation between foraging trips and nest attendance shifts (the duration of which is termed “shift duration”). A foraging trip begins with the first wet period after a >12‐h dry period and ends at the start of the next >12‐h dry period. The term “individual trip duration” is applied to the length of a foraging trip of a focal bird. During incubation and for a month after hatching, albatross parents never leave their offspring unattended (Weimerskirch et al., 2000; Weimerskirch & Lys, 2000). As one parent's presence on land indicates that their partner is at sea, the individual trip durations of the birds without loggers were estimated from the shift durations of their monitored mates. Partner foraging trips were defined as the time between the end of the tagged bird's foraging trip and the start of the tagged bird's next foraging trip. The observed and predicted partner foraging trip durations were compared in three pairs where both partner's carried loggers and the average accuracy were found to be 93.80% (± SD = 5.40%). A delay in the nest‐bound bird's departure after their partner's return may cause a period of overlap at the nest. The potential impact of this was assessed using the pairs where both members carried loggers (*n* = 3). The average overlap at the nest was 1 h 53 min, 1.40% of the average incubation trip duration and 2.88% of the average brooding trip duration (Appendix S1).

#### 2.3.2 Separation of breeding stages

In our dataset, mean incubation trip durations (5.60 ± 4.28 SD days) were more than twice as long as mean brooding trip durations (2.72 ± 1.36 SD days). The differences in breeding behavior between incubation and brooding lead to significant changes in trip duration (Appendix S2), so data from these breeding stages were analyzed in two separate models. Individual lay and hatch dates were not available; however, this species is known to display remarkable consistency in their phenology (Fressanges du Bost & Segonzac, 1976; Jones et al., 2017; Weimerskirch, 1992). Incubation was therefore assumed to begin on 16th December (the earliest possible lay date) (Fressanges du Bost & Segonzac, 1976; Weimerskirch, 1995). Where possible, hatch date was determined for each pair separately by observing a sudden drop in trip duration (*n* = 66) (Appendix S2). Where this was unclear, the average hatch date of 15th March was applied (*n* = 5). We constrained the end of brooding to 11th April based on previous publications (Fressanges du Bost & Segonzac, 1976; Weimerskirch et al., 2000) and on the data from the pairs where both individuals were tagged (Appendix S1).

#### 2.3.3 Creation of coordination variable

**FIGURE 1:**
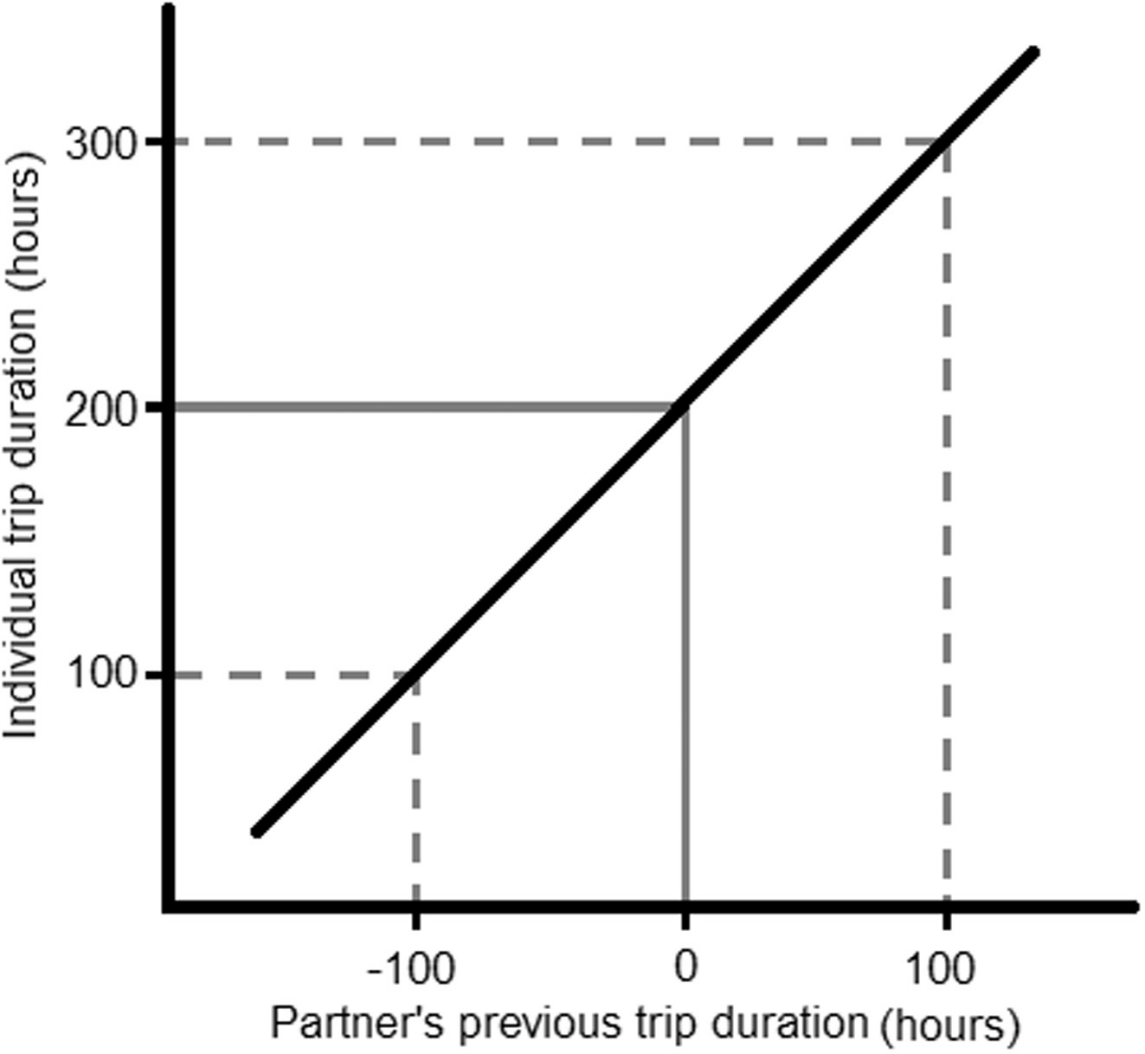
Demonstration of how the relationship between individual trip duration and partner's previous trip duration is indicative of coordination. In this hypothetical scenario, a pair of birds are exhibiting perfect coordination (slope represented by the black line). The partner bird's average trip duration is 200 h. Because there is perfect coordination within the pair, when the partner birds behave according to this average, the focal bird matches this trip duration exactly (solid gray line). In the event that the partner bird takes a foraging trip with is longer or shorter than average (e.g., ±100 h, dashed gray lines), we would expect the focal bird to adjust their trip duration accordingly. Thus, a positive slope between individual trip duration and partner's previous trip duration suggests that the focal bird is responding to its partner's previous behavior and coordination exists within the pair.

To establish whether partners matched trip durations, partner behavior (hereafter “partner's previous trip duration”) was included in the model. This was calculated as the deviation of each observed partner trip duration from that partner's average previous trip duration during that breeding stage (Figure 1). This controlled for each partner's broader trip duration pattern and attempted to measure if focal birds were responding only to their partner's most recent trip duration. The first brooding trip duration for each pair was excluded from the brooding model, because the associated partner's previous trip duration observation occurred during incubation. A total of 260 incubation trip durations from 7 years were included in the analysis (2008: *n* = 10, 2009: *n* = 70, 2010: *n* = 49, 2011: *n* = 38, 2012: *n* = 14, 2013: *n* = 38, 2014: *n* = 41), while 611 brooding trip durations from 6 years were included in the brooding analysis (2008: *n* = 137, 2009: *n* = 174, 2010: *n* = 70, 2011: *n* = 81, 2012: *n* = 84, 2013: *n* = 65).

#### 2.3.4 Statistical analysis

Two linear mixed models were constructed, one for incubation and one for brooding. All analyses were performed in R 4.0.3 (R Core Team, 2021) using lme4 (Bates & Maechler, 2010).

Individual trip duration (h) was square root transformed to correct for positive skew and included as the response variable. Focal boldness, partner boldness, and partner's previous trip duration (to measure coordination) were fitted as continuous fixed effects, while sex and new partnership (either breeding together for the first time that year or had bred together in at least one previous year) were fitted as binary fixed effects (Patrick et al., 2020). Focal age and partner age were fitted as both linear and quadratic effects. An investigation into the potential effects of multicollinearity found that the simultaneous inclusion of both focal and partner variables had no impact on model outcome (Appendix S3). The date (the number of days since 16th December) was included as a fixed factor to control for the temporal changes within breeding stages.

The slope of the relationship between individual trip duration and partner's previous trip duration was used as a measure of coordination strength. A positive slope was indicative of coordination (Figure 1). Interactions between partner's previous trip duration and focal age (linear and quadratic), partner age (linear and quadratic), focal boldness, partner boldness, and new partnership were fitted. Should any of these interactions appear to be important, this would suggest that the relevant intrinsic variable has influence over the slope between individual foraging trip duration and partner's previous trip duration. As this slope represents coordination strength, this would imply that the intrinsic variable affects variation in coordination strength between pairs. Interactions between focal age and partner age (linear and quadratic) and their equivalent boldness variable (focal boldness or partner boldness) were also fitted (Patrick & Weimerskirch, 2015). Interactions between focal boldness and sex, and partner boldness and sex were also included in both models (Appendix S3). Pair ID and year were fitted as random intercepts and partner's previous trip duration was included as a random slope, so that the strength of coordination could vary between pairs. All explanatory variables were scaled (mean 0 ± 1 SD) prior to the separation of breeding stages, so that the model coefficients would be directly comparable between breeding stages.

Goodness‐of‐fit measures for the global models (marginal and conditional *R* ^2^) were calculated using the MuMin package (Bartoń, 2020) and reported in accordance with Nakagawa and Schielzeth (2013). Akaike's information criterion, adjusted to account for a small sample size (AIC_C_), was applied during model selection. Following the construction of the global models, all possible models were generated and ranked by AIC_c_ score. A group of best‐fitting models with ΔAIC_c_ < 2 was then extracted. Nested models (more complex versions of simpler models with a lower AIC_c_) were excluded to improve inference (Arnold, 2010; Harrison et al., 2018; Richards et al., 2011). If multiple models were included in the best‐fitting set (Appendix S4), model averaging was applied to obtain new parameter estimates. Due to concerns previously raised about model averaging interaction terms (Cade, 2015), averaged coefficients of retained interactions and associated fixed effects are not reported, and instead all coefficients from the top model set can be found in the supplementary material (Appendix S4).

### 2.4 Ethics

All field procedures were approved by the Ethics Committee of L'Institut Polaire Français Paul‐Emile Victor (IPEV).

## 3 RESULTS

### 3.1 Intrinsic variables and individual trip duration

Several intrinsic variables impacted individual trip duration and the retention of these variables varied between breeding stages (Table 1).

**TABLE 1:** Averaged parameter estimates and standard errors from the best supported models (non‐nested models with Δ Akaike's information criterion_c_ < 2) investigating the impact of intrinsic variables on foraging trip duration and parental care coordination during incubation and brooding.

	Incubation			Brooding		
	Retained in final model	Model averaged estimate	Standard error	Retained in final model	Model averaged estimate	Standard error
Effects on individual shift duration						
Intercept	Y	14.82	0.82	Y	8.84	0.31
Focal age	N			N		
Focal age^2^	N			N		
Partner age	Y	1.31	2.38	N		
Partner age^2^	Y	−1.18	2.15	N		
Focal boldness	N			Y	NA	NA
Partner boldness	Y	−0.24	0.33	Y	−0.24	0.10
Date	Y	2.54	0.48	Y	−1.22	0.42
New partner—true	N			Y	0.41	0.37
Partner's previous trip duration	Y	0.19	0.19	Y	0.18	0.16
Sex—male	Y	−1.39	0.52	Y	NA	NA
Interactions acting on individual shift duration						
Focal boldness × focal age	N			N		
Focal boldness × focal age^2^	N			N		
Focal age × partner's previous trip duration	N			N		
Focal age^2^ × partner's previous trip duration	N			N		
Focal boldness × partner's previous trip duration	N			N		
New partner‐true × partner's previous trip duration	N			N		
Partner boldness × partner age	N			N		
Partner boldness × partner age^2^	N			N		
Partner age × partner's previous trip duration	N			N		
Partner age^2^ × partner's previous trip duration	N			N		
Partner boldness × partner's previous trip duration	N			N		
Focal boldness × sex—male	N			Y	NA	NA
Partner boldness × sex—male	N			N		

*Note*: Square root transformed foraging trip length (h) was modeled as the response variable. All continuous variables were scaled (mean = 0 ± 1 SD) prior to separating the breeding stages. Year and pair ID were fitted as random intercepts, and partner's previous trip duration was fitted as a random slope in all models. Averaged coefficients of retained interactions and associated fixed effects are not reported (marked “NA”), but the full best supporting models can be found in the supplementary material (Appendix S4).

#### 3.1.1 Incubation

**FIGURE 2:**
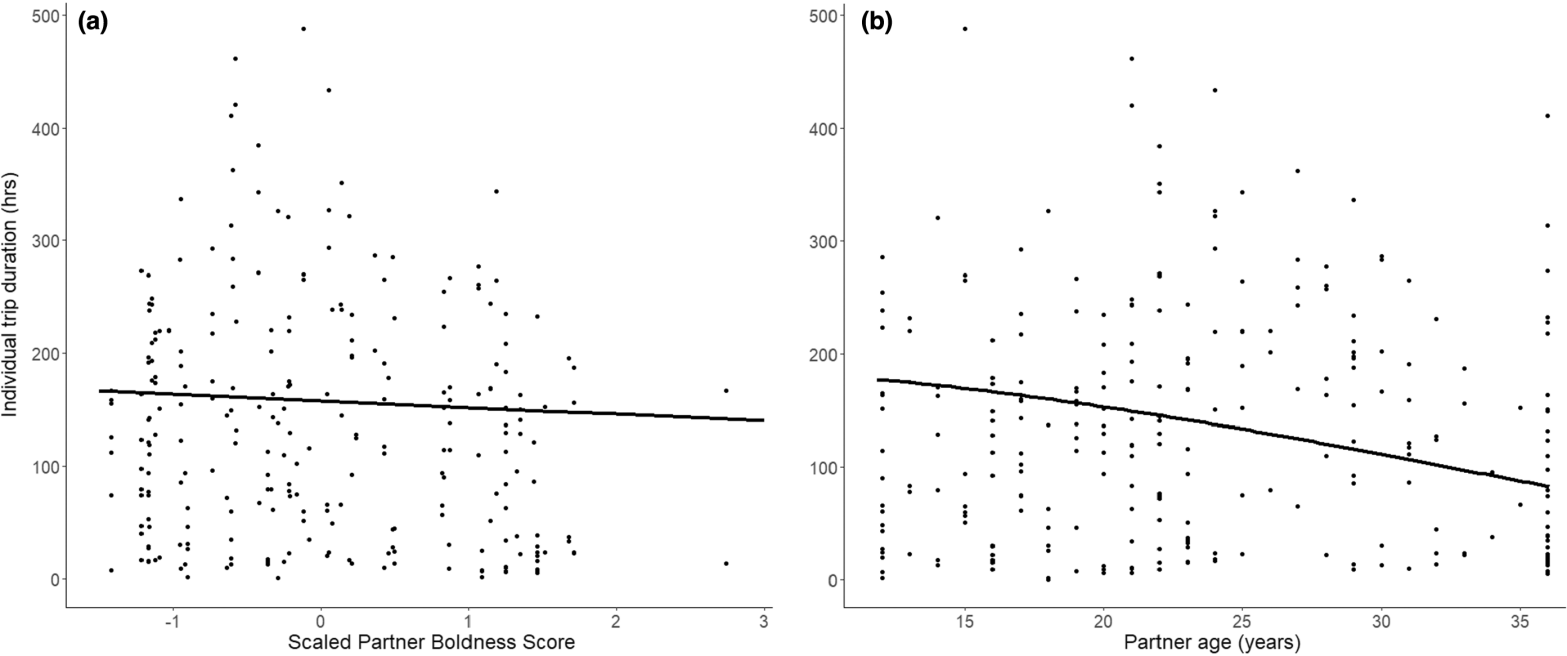
The effects of (a) partner boldness score and (b) partner age on individual trip duration during incubation. Partner boldness scores are scaled (M = 0, SD = 1). Birds with larger scores are considered to be bolder than those with lower scores. Partner age is presented unscaled. The mild negative slope of partner boldness score indicates that birds with bolder partners take shorter foraging trips. The weak, negative quadratic relationship between individual trip duration and partner age suggests that birds with older partners take shorter foraging trips.

Following model selection based on the incubation global model (marginal *R* ^2^ = .26, conditional *R* ^2^ = .38), sex was retained as an important influence on trip duration, with males taking shorter foraging trips (mean = 121.62 h ± 103.97 SD) than females (mean = 147.33 h ± 100.00 SD) (Table 1). Partner boldness, but not focal boldness was also retained in the incubation model, suggesting that birds with bolder partners engage in shorter foraging trips (Table 1, Figure 2a). Partner, but not focal age was found to have a weak, negative quadratic effect on individual trip duration (Table 1, Figure 2b) suggesting birds with older partners made shorter foraging trips.

#### 3.1.2 Brooding

**FIGURE 3:**
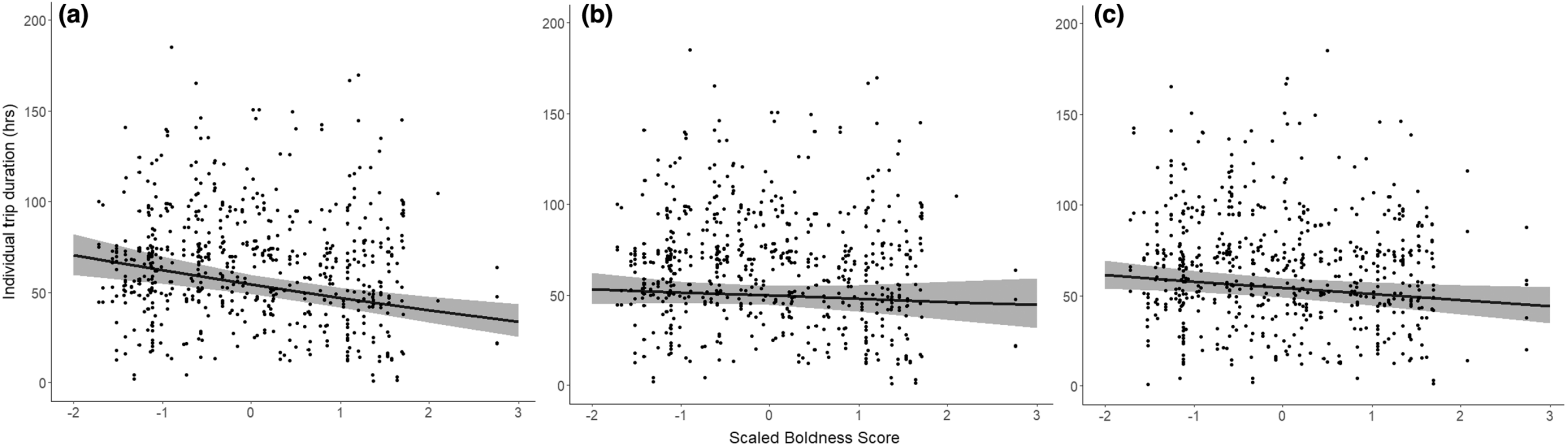
The relationship between (a) female bird focal boldness score, (b) male bird focal bird score, and (c) partner bird boldness score and individual trip duration during brooding. Boldness scores are scaled (M = 0, SD = 1). One outlier individual trip duration (327 h) has been removed to improve clarity. Shaded areas represent 95% confidence intervals. Birds with larger scores are considered to be bolder than those with lower scores. The steeper negative slope for female focal boldness scores suggests that the decline in trip duration with increased boldness is stronger in females. The negative slope of partner boldness score indicates that birds with bolder partners take shorter foraging trips.

The brooding global model (marginal *R* ^2^ = .11, conditional *R* ^2^ = .21) was refined via the model selection process. In contrast to incubation, focal boldness was retained in the top model set during brooding, as was the interaction between focal boldness and sex (Table 1, Figures 3a,b, Appendix S4). Bolder birds took shorter foraging trips overall; however, the negative effect of focal boldness score on foraging trip duration was slightly stronger in females (Figure 3a) than in males (Figure 3b). As in incubation, male birds (male mean = 62.49 h ± 28.06 SD, female mean = 68.11 h ± 36.37 SD) and those with bolder partners were found to engage in shorter trips during brooding (Table 1, Figure 3c). Brooding birds in new partnerships made longer foraging trips (mean = 75.41 h ± 48.84 SD) than those in established pairs (mean = 64.01 h ± 29.67 SD). Neither focal age nor partner age was found to impact individual trip duration during brooding (Table 1).

### 3.2 Intrinsic traits and coordinated parental care

**FIGURE 4:**
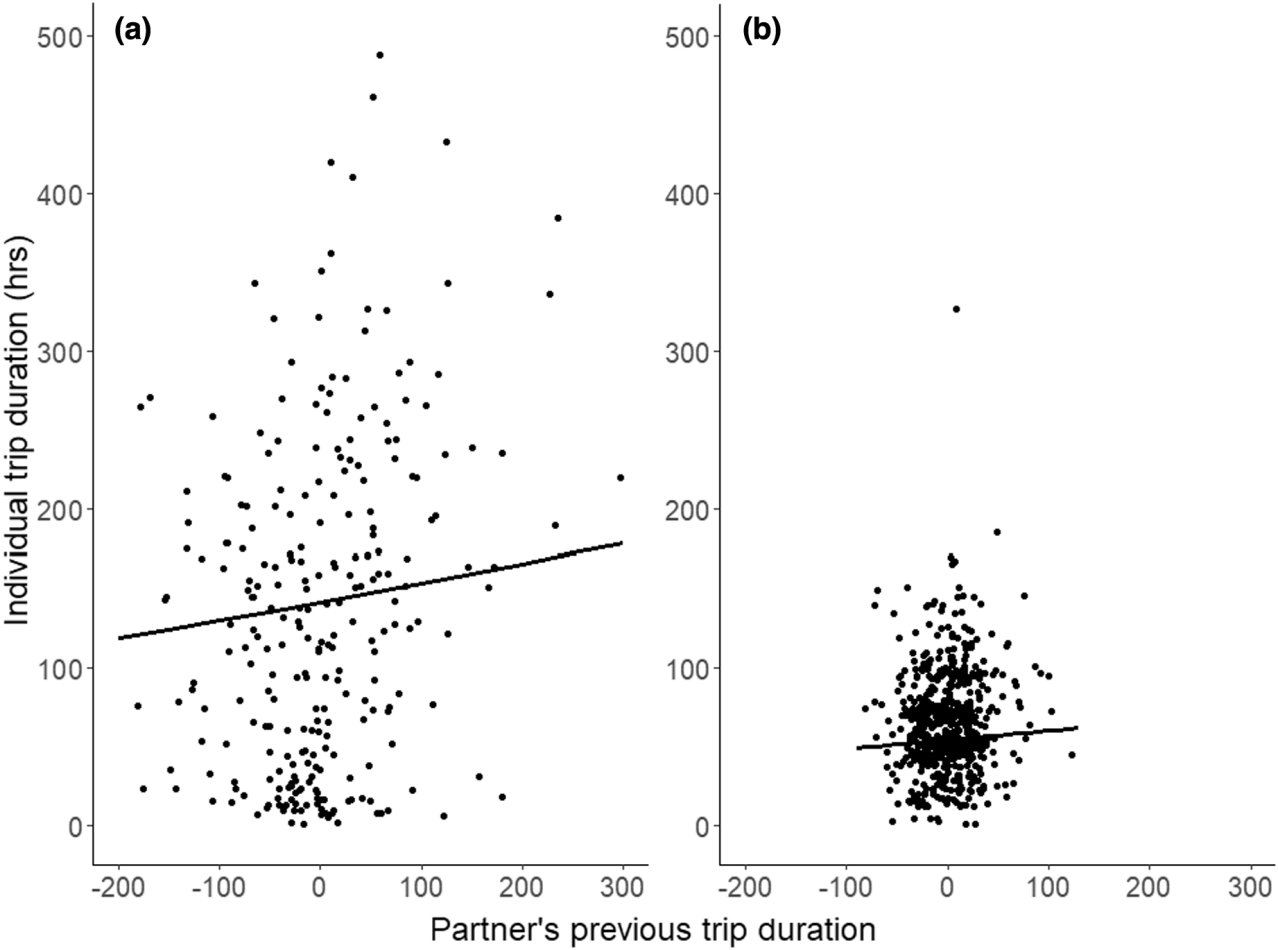
The relationship between individual trip duration (h) and partner's previous trip duration (the deviation of the partner's last trip duration from their average trip duration) (h) in (a) incubation and (b) brooding. The angle of the slope (represented by the regression line) indicates strength of coordination, suggesting that it is of approximately equal strength in both incubation (0.19) and brooding (0.18) within the population.

Partner's previous trip duration was retained as an influence on individual trip duration for both breeding stages. The average best‐fitting model's slope of partner's previous trip duration was similar in incubation (0.19, SE = 0.19) (Table 1, Figure 4a) when compared with brooding (0.18, SE = 0.16) (Table 1, Figure 4b), suggesting that at the birds are exhibiting a mild degree of coordination within the population and that this coordination is equally strong in both breeding stages. The models displayed little variation in coordination strength (Appendix S5), and there was no interactive effect of partner's previous trip duration with any of the focal bird or partner bird intrinsic variables in either incubation or brooding (Table 1). This suggests that interpair variation in coordination strength was limited in this species and that the variation which was detected was not influenced by any of the intrinsic variables included in this study.

## 4 DISCUSSION

Our investigation into the impacts of pair member intrinsic characteristics on the individual trip durations of breeding albatrosses found that: (1) Partner intrinsic traits had a greater impact on individual trip duration than expected; and (2) the influential variables varied depending on breeding stage. Increased partner boldness was linked to shorter foraging trips in both incubation and brooding, while increased partner age was linked to shorter trips in incubation only. Although males made shorter trips overall, during brooding, the negative relationship between trip duration and focal boldness was marginally stronger in females. Brooding birds from new partnerships spent longer at sea than those from established pairs.

Regardless of breeding stage, we report that albatrosses appear to coordinate parental care and that at the population level, this coordination was of equal strength in the incubation and brooding periods. Partner's previous trip duration was retained in both the incubation and brooding models as an influence on individual trip duration; however, there was only limited variation in slope between pairs suggesting that there was little interpair variation in coordination strength. Furthermore, there were no interactive effects between this variable and any of the intrinsic variables (sex, age, boldness, new partnership) of either the focal or partner bird. These results suggest that although the intrinsic traits of pair members may impact their individual foraging decisions, they do not affect how the birds respond to their partner's behavior.

### 4.1 Effect of intrinsic variables on individual trip duration

The importance of partner intrinsic traits in determining trip duration was contrary to our prediction that the focal bird's traits would be the primary drivers of this foraging behavior. In systems with obligate biparental care, information transfer relating to each individual's ability to contribute should play a crucial role in the optimization of parental investment (Griffith, 2019; Roughgarden, 2012). In albatrosses, the foraging bird dictates the cost to its nest‐bound partner through the length of its foraging trip. If a partner bird's intrinsic state is connected to their ability to maintain their body condition above the threshold for desertion (Weimerskirch, 1995), the foraging bird could use this information to judge their partner's willingness or capacity to care. This would allow them to adjust foraging trip duration appropriately, for example, by preventing over‐long absences to minimize desertion risk. Some seabird species are thought to adjust their behavior based on their perception of their partner's body condition (Gillies et al., 2021; Takahashi et al., 2017); however, our results suggest that other, more stable partner traits (i.e., boldness and age) may also provide insight into partner state, allowing foraging birds to make more informed choices at sea.

Not all of our observed results are in keeping with current theory, particularly our finding that birds with bolder partners made shorter foraging trips. Theory predicts that a shorter lifespan should cause bolder individuals to invest more heavily in their current brood, rather than conserve resources for future breeding opportunities (Réale et al., 2010; Wolf et al., 2007). It is plausible that a stronger immediate commitment in this species could manifest as a higher threshold for desertion, as to abandon might incur greater fitness costs for bold birds compared to shyer conspecifics (Cole & Quinn, 2014; Patrick & Weimerskirch, 2015). We would therefore expect birds mated to bolder partners to lengthen their foraging trips in response to a lower desertion risk; however, we report the opposite. Although some albatrosses may be predisposed to abandon (Weimerskirch, 1992), the impact of personality on desertion risk has never been directly explored in this species. Our results suggest more detailed examination is required to explore this unexpected relationship and gain a greater understanding of how bold individuals allocate their parental investment.

In contrast, our finding that bolder birds and those with bolder partners take shorter foraging trips during brooding corresponds more closely with the literature. Weimerskirch and Lys (2000) described how shorter foraging trips led to an increase in chick provisioning at the expense of the parent's body mass. Bolder individuals may increase immediate investment by shortening trip duration, thereby reducing the resources available to any future offspring. Sex is thought to mediate personality effects in multiple avian species (e.g., Patrick & Weimerskirch, 2014; Schuett & Dall, 2009; Traisnel & Pichegru, 2019). Our results reiterate the well‐established pattern that male albatrosses engage in longer incubation bouts and shorter foraging trips (Weimerskirch et al., 2000; Weimerskirch & Lys, 2000); however, we also report that the boldest females tended to have shorter brooding trips than the boldest males. A larger body size may allow males to protect their condition during incubation (Croxall & Ricketts, 1983) and carry heavier meals when provisioning (Weimerskirch & Lys, 2000). If sexual dimorphism constrains female provisioning capability in terms of meal size, perhaps the boldest females instead boost current investment by shortening foraging trip durations to increase provision rate.

The tendency of new partnerships to take longer brooding trips may be connected to reports that new pairs often suffer from a lack of mate familiarity (Bried et al., 2003; Jouventin et al., 1999) or experience (Jones et al., 2014; Weimerskirch, 1992). Although albatrosses mate assortatively according to age (Jouventin et al., 1999; Appendix S3), previous work on a potential link between age and trip duration in this population is inconclusive (Lecomte et al., 2010; Patrick & Weimerskirch, 2015). Aging male albatrosses appear to make foraging decisions aimed at reducing energy expenditure (Lecomte et al., 2010; Weimerskirch et al., 2014), while Clay et al. (2018) found that older individuals suffered fitness consequences following a sabbatical year requiring high foraging effort. If older individuals are more vulnerable to the long‐term consequences of overexertion or have lower desertion thresholds, it is possible that their partners, either through mate familiarity (Froy et al., 2013) or some other unknown mechanism, are able to recognize their partner's limitations. Responding by shortening their own foraging trips may limit the damage to their partner's condition and reduce the risk of desertion.

A change in focus from protecting pair collective fitness during incubation, to balancing parental investment during brooding may help to explain the absence of focal and partner age from the brooding results. Frankish et al. (2020) suggested that both black‐browed albatrosses and gray‐headed albatrosses *Thalassarche chrysostoma* were more capable of adjusting their foraging trip lengths according to age when they were not constrained by the demands of central place foraging. The flexibility provided during incubation may permit birds paired to older partners to adjust their trip duration more easily. Brooding parents lose mass extensively (Ricklefs, 1983; Weimerskirch et al., 1993), and as time constraints prevent them from acting to stabilize or improve their own condition (Weimerskirch & Lys, 2000), it is likely that they cannot make allowances to protect their mate. Furthermore, as the risk of partner desertion is greatly reduced during brooding (Weimerskirch, 1995), such action may be less necessary.

Our finding that individual foraging decisions are influenced by both focal and partner traits has implications for future studies focusing on parental behavior in all species which share care. Our results support the suggestion that pair members should be viewed as interdependent components of a single unit with a shared objective (Griffith, 2019; Roughgarden, 2012). As individual behavior could be considered to be a product of this unit, we argue that the traits and behavior of both pair members should be considered when attempting to interpret individual breeding behavior. In practice, we suggest that variables relating to both pair members be fitted in future models, as it is possible that the breeding behavior of individuals is best understood when viewed in the context of the pair as a whole.

### 4.2 Patterns of coordinated care

Our findings suggest that coordination strength is approximately equal in incubation and brooding when examined at the population level. Given the different constraints in the two breeding stages, it is plausible that the benefits of a coordinated schedule change over time. During incubation, when nest shift duration is highly variable at the population level (between 2 and 30 days) (Brown & Adams, 1984), coordination may help maintain both parents' long‐term condition by preventing prolonged incubation bouts. This simultaneously decreases the probability of reaching the critical threshold for desertion (Weimerskirch, 1995). In contrast, brooding nest shift durations are less variable and less likely to result in desertion (Weimerskirch et al., 2014; Weimerskirch & Lys, 2000). Coordination may therefore be repurposed to ensure regular chick provisioning (Grissot et al., 2019; Welcker et al., 2009). Previous work which has investigated the importance of coordination patterns in determining reproductive outcomes in seabirds has yielded mixed results (Grissot et al., 2019; Kavelaars et al., 2019; Wojczulanis‐Jakubas et al., 2018); however, no studies have specifically investigated wandering albatrosses. As our data included only a tiny number of failed breeding attempts (*N* = 4), we did not have enough variation in breeding success within our sample to establish if a link exists within this species. Therefore, our findings are only representative of successful pairs in this population; however, an investigation into the fitness consequences of coordination featuring a different sample of birds would be a logical path for future work.

We found no evidence that sex, focal age, partner age, focal boldness, partner boldness, or partnership status impacted coordination in either the incubation or brooding phases. This suggests that coordination functions independently of these individual intrinsic differences throughout the featured portion of the species' breeding cycle. Given that less interpair variation in coordination strength was observed than anticipated (Appendix S5), it is possible that an optimal coordination strategy exists in this species which allows pairs to simultaneously accomplish all their required foraging tasks and balance the constraints of relieving their partner alongside potential unknown, extrinsic factors (Patrick et al., 2020; Wojczulanis‐Jakubas et al., 2018). Additional work is required to explore what other drivers might be acting on albatross coordination patterns. Accounting for the role that environmental conditions may play in coordination is critical, as although pair members may make foraging decisions independently, if these are based on information from a shared environment (i.e., exposure to current weather conditions), they may make similar choices, thereby displaying foraging trips of equivalent length (Ihle et al., 2019; Santema et al., 2019). As the Crozet albatross population are sexually segregated when foraging (Weimerskirch et al., 1993, 2014), we are confident that shared environmental conditions are unlikely to be driving the coordination observed in our results. The most robust way to account for shared environmental conditions is to include these variables in the analysis, and so future work on similar study systems should aim to incorporate environmental variables directly into the models to help counteract any uncertainty (Santema et al., 2019; Schlicht et al., 2016).

In conclusion, although many questions remain surrounding the phenomenon of coordinated parental care, this work provides further evidence that both the role of the individual and the interactions within the pair are instrumental in determining collective parental care behavior. Such insight is vital when studying any system in which parents share care, if we are to fully comprehend how sexual conflict is resolved and how cooperation is preserved in long‐lived, monogamous species.

## AUTHOR CONTRIBUTIONS

**Fionnuala R. McCully:** Conceptualization (equal); formal analysis (lead); investigation (equal); project administration (equal); visualization (lead); writing – original draft (lead); writing – review and editing (lead). **Henri Weimerskirch:** Conceptualization (equal); data curation (lead); funding acquisition (lead); investigation (equal); methodology (lead); writing – original draft (supporting); writing – review and editing (equal). **Stephen J. Cornell:** Conceptualization (equal); formal analysis (supporting); investigation (equal); supervision (supporting); writing – original draft (supporting); writing – review and editing (equal). **Ben J. Hatchwell:** Conceptualization (equal); investigation (equal); supervision (supporting); writing – review and editing (equal). **Milena Cairo:** Conceptualization (equal); data curation (supporting); formal analysis (supporting); investigation (equal); writing – original draft (supporting); writing – review and editing (equal). **Samantha C. Patrick:** Conceptualization (equal); formal analysis (supporting); investigation (equal); methodology (supporting); supervision (lead); visualization (supporting); writing – original draft (supporting); writing – review and editing (equal).

## CONFLICT OF INTEREST

The authors declare there are no conflicts of interest.

### OPEN RESEARCH BADGES

This article has earned an Open Data badge for making publicly available the digitally‐shareable data necessary to reproduce the reported results. The data are available at https://github.com/fmccully/Waal‐Intrinsic‐Project.

## Supporting information


Appendix S1–S5

## Data Availability

Data and R code are available via Dryad: https://doi.org/10.5061/dryad.q573n5tnm.
