# Supplementary material for: Partner intrinsic characteristics influence foraging trip duration, but not coordination of care in wandering albatrosses Diomedea exulans
Source: Ecol Evol. 2022 Dec 15;12(12):e9621. doi: 10.1002/ece3.9621 (PMC9754911; doi:10.1002/ece3.9621)
Supplement: Supplementary file 1 — Appendix S1–S5 [file ECE3-12-e9621-s001.docx]

Partner intrinsic characteristics influence foraging trip duration, but not coordination of care in wandering albatrosses *Diomedea exulans*

**Supplementary material**

**Appendices**

Appendix S1: observed and predicted shift duration comparison

The method used to estimate partner trip durations was tested for accuracy using the three pairs where both pair members were tagged. The observed trip duration (calculated from the bird’s data logger) was compared to the predicted trip duration (estimated from its partner’s behaviour) and the deviation was calculated. A graphical representation of the results from an anonymised example bird (22566) is provided (Fig. S1).


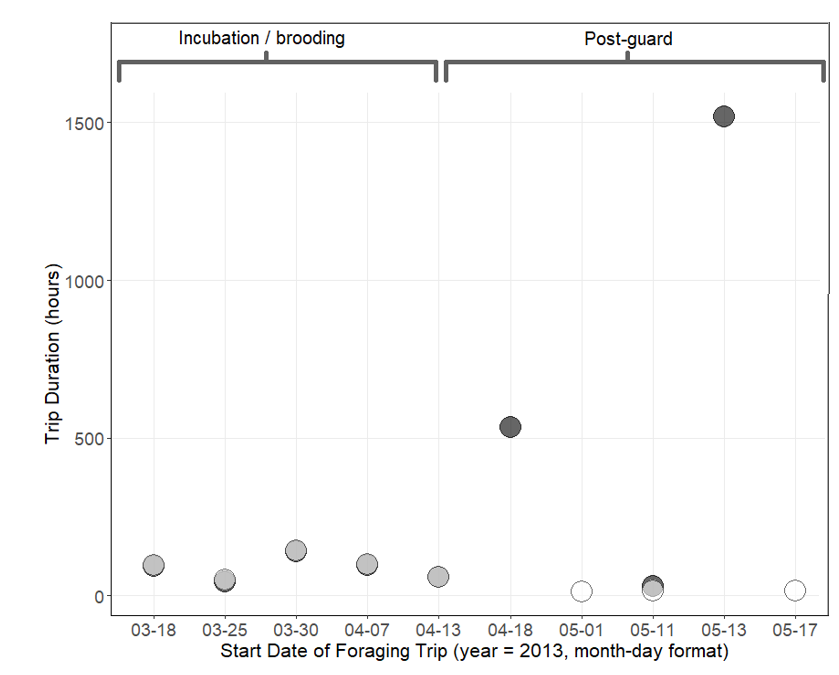


Fig. S1 Comparison of observed (black) and predicted (white) trip duration values for the bird 22566. After mid-April, the bird enters post-guard and the method for predicting its trip durations becomes unreliable as the chick no longer needs constant parental attendance at the nest.

For this individual, predicted and original trip length are similar until 13^th^ April. Predicted trips deviated from original trips between 1.68-7.01% before this point. After this point the method did not correctly predict the dates of several following visits. This provided us with additional cause to set the end of the brooding period as 11^th^ April.

Appendix S2: separation of breeding stages

Most commonly, foraging trip duration becomes dramatically shorter at the start of brooding, and visual inspection of the data could be used to separating the incubation and brooding breeding stages. The anonymised pair 18897/20800 is provided as an example (Fig. S1). In such cases, the date of the last long trip duration was considered to be the end of incubation.


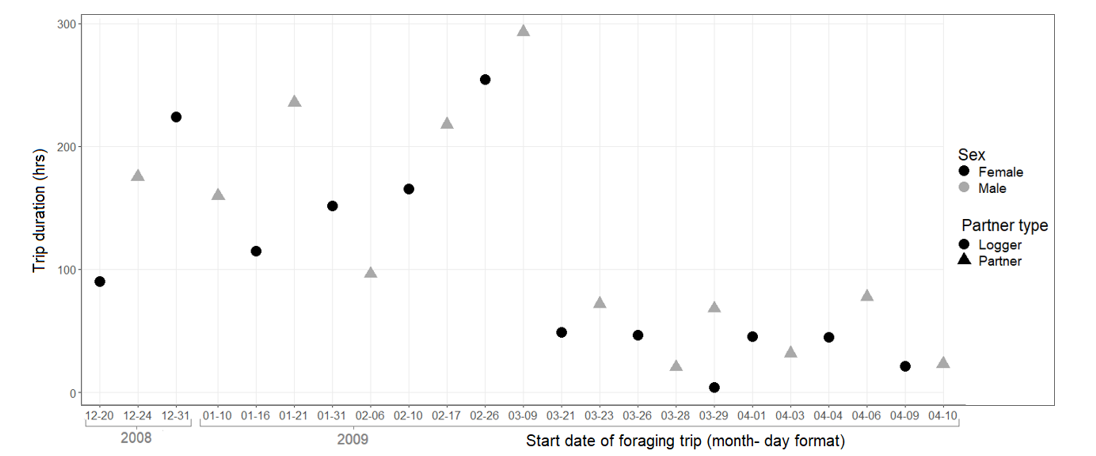


Fig. S1 Pair 18807/20800’s trip duration patterns showing a decline in trip duration after the 9^th^ March 2009 suggesting that brooding commenced after this date.

In five cases, the end of incubation could not be determined via visual inspection and so the average date of 15^th^ March was used to signify the transition into brooding. An anonymised example pair (25261/25251) is provided (Fig. S2).


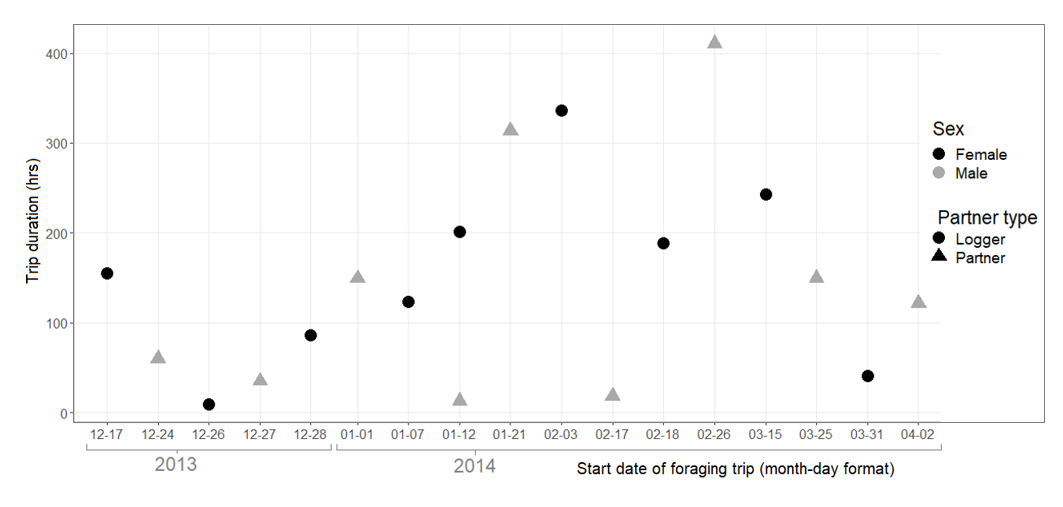


Fig. S2 Pair 25261/25251 trip duration patterns did not display a clear distinction between incubation and brooding and so 15^th^ March was selected as the end date for incubation.

Appendix S3: investigation to importance of corresponding age and boldness variables in the top model sets

We addressed the possibility that any correlations which exist between focal bird age and partner bird age, and focal bird boldness and partner boldness (Table S1) might impact the final models by creating exploratory models for both incubation (Tables S2-S4) and brooding (Tables S5-S7) which featured either focal bird variables or partner bird variables. In all cases, once the model averaging procedure was completed, only those variables which were retained in the models featured in this paper’s main body were retained, suggesting that the presence of their equivalent variable has no bearing on the overall result (Table S4 & S7). We also used these models to test the validity of including interactions between sex and age (focal and partner) and boldness (focal and partner) in the models. This evidence was then applied to reduce the number of interactions in the models to prevent over-complexity.

Table S1. Pearson's product-moment correlation coefficients of the relationships between focal age and partner age, and focal boldness and partner boldness in the incubation and brooding data sets.

|  | Incubation | Brooding |
| --- | --- | --- |
| Age | 0.67 | 0.59 |
| Boldness | -0.51 | -0.05 |

Table S2. Table of coefficients generated by the top model set (including nested models) derived from the incubation global model with partner age and boldness variables excluded. All continuous variables were scaled (mean = 0 ± 1 SD) prior to separating the breeding stages. Year and pair ID were fitted as random intercepts, and partner’s previous trip duration was fitted as a random slope in all models.

| Model No. | Intercept | Date | Partner's previous trip duration | Sex-male | Focal boldness |
| --- | --- | --- | --- | --- | --- |
| 105 | 14.84 | 2.43 | 0.30 | -1.60 |  |
| 73 | 15.17 | 2.72 | - | -1.61 |  |
| 121 | 14.74 | 2.41 | 0.31 | -1.48 | 0.16 |

In incubation, only date, sex and partner’s previous trip duration were eventually retained (Table S4). Focal boldness (Table S2) was eliminated with the nested models, as occurred during the results presented in the main body.

Table S3. Table of coefficients generated by the top model set (including nested models) derived from the incubation global model with focal age and boldness variables excluded. All continuous variables were scaled (mean = 0 ± 1 SD) prior to separating the breeding stages. Year and pair ID were fitted as random intercepts, and partner’s previous trip duration was fitted as a random slope in all models.

| Model no. | Intercept | Date | Partner  boldness | Partner previous  trip duration | Sex-male | Partner  age | Partner  age^2^ | Partner  age^2^ x partner  boldness | Partner  age x partner boldness | Partner  age x sex –male | Partner  age^2^ x sex­–male | Partner boldness x partner's  previous  trip duration |
| --- | --- | --- | --- | --- | --- | --- | --- | --- | --- | --- | --- | --- |
| 115 | 14.72 | 2.48 | -0.52 | 0.30 | -1.21 |  |  |  |  |  |  |  |
| 127 | 14.44 | 2.39 | -0.45 | 0.31 | -1.16 | 4.18 | -3.74 |  |  |  |  |  |
| 2175 | 14.41 | 2.37 | -0.24 | 0.31 | -1.17 | 4.99 | -4.43 | -0.36 |  |  |  |  |
| 111 | 11.52 | 2.34 |  | 0.31 | -1.48 | 4.65 | -4.20 |  |  |  |  |  |
| 83 | 15.06 | 2.77 | -0.53 |  | -1.21 |  |  |  |  |  |  |  |
| 99 | 14.84 | 2.43 |  | 0.30 | -1.60 |  |  |  |  |  |  |  |
| 383 | 14.40 | 2.38 | -0.23 | 0.31 | -1.15 | 4.99 | -4.42 |  | -0.39 |  |  |  |
| 9343 | 14.59 | 2.42 | -0.54 | 0.30 | -0.31 | 0.62 | -0.52 |  |  | 7.18 | -6.37 |  |
| 95 | 14.77 | 2.68 | -0.46 |  | -1.16 | 4.06 | -3.63 |  |  |  |  |  |
| 16499 | 14.72 | 2.48 | -0.52 | 0.30 | -1.21 |  |  |  |  |  |  | 0.11 |
| 2143 | 14.76 | 2.67 | -0.25 |  | -1.18 | 4.84 | -4.30 | -0.35 |  |  |  |  |
| 79 | 14.85 | 2.63 |  |  | -1.50 | 4.53 | -4.09 |  |  |  |  |  |
| 67 | 15.17 | 2.72 |  |  | -1.61 |  |  |  |  |  |  |  |
| 3199 | 14.49 | 2.37 | -0.20 | -0.31 | -1.33 | 5.19 | -4.89 | -0.46 |  | 0.54 |  |  |

In contrast, more fixed effect and interaction terms appeared in the top model set when the partner age and boldness variables are included (Table S3). Both partner age and partner boldness feature in the best fitting models, and are retained in the final model averaged coefficient (Table S4).

Table S4. Averaged parameter estimates and standard errors from the best supported models (non-nested models with Δ Akaike’s information criterion _c_ < 2) created to investigate the impact of intrinsic variables on parental care coordination during incubation with partner bird age and boldness, and focal age and boldness excluded respectively. Excluded variables are marked ‘-’. All continuous variables were scaled (mean = 0 ± 1 SD) prior to separating the breeding stages. Year and pair ID were fitted as random intercepts, and partner’s previous trip duration was fitted as a random slope in all models.

|  | Focal bird variables only | | | Partner bird variables only | | |
| --- | --- | --- | --- | --- | --- | --- |
|  | Retained in final model | Model averaged estimate | Standard error | Retained in final model | Model averaged estimate | Standard error |
| Effects on individual shift duration |  |  |  |  |  |  |
| Intercept | Y | 14.97 | 0.81 | Y | 14.82 | 0.82 |
| Focal age | N |  |  | - |  |  |
| Focal age^2^ | N |  |  | - |  |  |
| Partner age | - |  |  | Y | 1.31 | 2.38 |
| Partner age^2^ | - |  |  | Y | -1.18 | 2.15 |
| Focal boldness | N |  |  | - |  |  |
| Partner boldness | - |  |  | Y | -0.24 | 0.33 |
| Date | Y | 2.55 | 0.47 | Y | 2.54 | 0.48 |
| New partner ­–true | N |  |  | N |  |  |
| Partner’s previous trip duration | Y | 0.19 | 0.19 | Y | 0.19 | 0.19 |
| Sex ­–male | Y | -1.60 | 0.47 | Y | -1.39 | 0.52 |
| Interactions acting on individual shift duration |  |  |  |  |  |  |
| Focal boldness x focal age | N |  |  | - |  |  |
| Focal boldness x focal age^2^ | N |  |  | - |  |  |
| Focal age x partner’s previous trip duration | N |  |  | - |  |  |
| Focal age^2^ x partner’s previous trip duration | N |  |  | - |  |  |
| Focal boldness x partner’s previous trip duration | N |  |  | - |  |  |
| New partner-true x partner’s previous trip duration | N |  |  | N |  |  |
| Partner boldness x partner age | - |  |  | N |  |  |
| Partner boldness x partner age^2^ | - |  |  | N |  |  |
| Partner age x partner’s previous trip duration | - |  |  | N |  |  |
| Partner age^2^ x partner’s previous trip duration | - |  |  | N |  |  |
| Partner boldness x partner’s previous trip duration | - |  |  | N |  |  |
| Focal boldness x sex ­–male | N |  |  | - |  |  |
| Partner boldness x sex –male | - |  |  | N |  |  |
| Age x sex ­–male | N |  |  | - |  |  |
| Age2 x sex ­–male | N |  |  | - |  |  |
| Partner age x sex –male | - |  |  | N |  |  |
| Partner age^2^ x sex ­–male | - |  |  | N |  |  |

Table S5. Table of coefficients generated by the top model set (including nested models) derived from the brooding global model with partner age and boldness variables excluded. All continuous variables were scaled (mean =0 ± 1 SD) prior to separating the breeding stages. Year and pair ID were fitted as random intercepts, and partner’s previous trip duration was fitted as a random slope in all models.

| Model no. | Intercept | New partnership  ­–true | Date | Focal  boldness | Partner's previous  trip duration | Sex­ ­male | Focal  boldness x sex ­ ­–male | Focal boldness x  partner's previous trip duration | New partnership ­–true x partner's previous trip duration |
| --- | --- | --- | --- | --- | --- | --- | --- | --- | --- |
| 8317 | 8.90 | 0.55 | -1.23 | -0.45 | 0.24 | -0.42 | 0.42 |  |  |
| 12413 | 8.92 | 0.56 | -1.26 | -0.45 | 0.27 | -0.42 | 0.41 | -0.19 |  |
| 8313 | 8.94 |  | -1.18 | -0.44 | 0.24 | -0.42 | 0.42 |  |  |
| 8285 | 8.98 | 0.57 | -1.36 | -0.45 |  | -0.42 | 0.41 |  |  |
| 12409 | 8.96 |  | -1.21 | -0.44 | 0.27 | -0.42 | 0.41 | -0.19 |  |
| 10365 | 8.90 | 0.56 | -1.20 | -0.45 | 0.20 | -0.42 | 0.42 |  | 0.29 |
| 14461 | 8.92 | 0.56 | -1.25 | -0.45 | 0.21 | -0.42 | 0.41 | -0.21 | 0.36 |
| 8281 | 9.02 |  | -1.31 | -0.44 |  | -0.42 | 0.41 |  |  |

Many variables which were retained in the final brooding model continue to be retained without the presence of those representing partner age and boldness, including focal boldness, new partnership and the interaction between focal boldness and sex (Table S7). As in the main results, focal age is notably absent (Table S5). These results provide compelling evidence to include a focal ‘boldness x sex’ interaction term in the main analysis.

Table S6. Table of coefficients generated by the top model set (including nested models) derived from the brooding global model with focal age and boldness variables excluded. All continuous variables were scaled (mean = 0 ± 1 SD) prior to separating the breeding stages. Year and pair ID were fitted as random intercepts, and partner’s previous trip duration was fitted as a random slope in all models.

| Model no. | Intercept | New  partnership –true | Date | Partner's previous  trip duration | Sex ­–male | Partner  boldness | Partner boldness x  partner previous trip duration | New partnership –true x  partner's previous shift duration |
| --- | --- | --- | --- | --- | --- | --- | --- | --- |
| 52 | 8.61 | 0.54 | -1.21 | 0.24 |  | -0.16 |  |  |
| 51 | 8.66 |  | -1.17 | 0.24 |  | -0.16 |  |  |
| 100 | 8.76 | 0.54 | -1.23 | 0.24 | -0.24 |  |  |  |
| 4148 | 8.60 | 0.54 | -1.19 | 0.23 |  | -0.16 | 0.16 |  |
| 99 | 8.80 |  | -1.19 | 0.24 | -0.24 |  |  |  |
| 20 | 8.69 | 0.55 | -1.34 |  |  | -0.16 |  |  |
| 36 | 8.64 | 0.54 | -1.24 | 0.23 |  |  |  |  |
| 4147 | 8.64 |  | -1.15 | 0.23 |  | -0.16 | 0.17 |  |
| 116 | 8.69 | 0.54 | -1.21 | 0.24 | -0.16 | -0.12 |  |  |
| 35 | 8.68 |  | -1.20 | 0.24 |  |  |  |  |
| 180 | 8.60 | 0.54 | -1.19 | 0.19 |  | -0.16 | 0.17 | 0.29 |
| 68 | 8.84 | 0.55 | -1.36 |  | -0.24 |  |  |  |
| 19 | 8.74 |  | -1.30 |  |  | -0.16 |  |  |
| 115 | 8.74 |  | -1.17 | 0.24 | -0.16 | -0.12 |  |  |
| 67 | 8.88 |  | -1.32 |  | -0.24 |  |  |  |
| 4212 | 8.68 | 0.53 | -1.19 | 0.23 | -0.16 | -0.12 | 0.16 |  |
| 4 | 8.72 | 0.55 | -1.37 |  |  |  |  |  |

The same variables are retained in the final brooding models (Table S7) suggesting that any relationship between age and partner age or between focal boldness and partner boldness has no bearing on the overall outcome of the analysis.

Table S7. Averaged parameter estimates and standard errors from the best supported models (non-nested models with Δ Akaike’s information criterion _c_ < 2) created to investigate the impact of intrinsic variables on parental care coordination during brooding with partner bird age and boldness, and focal age and boldness excluded respectively. Excluded variables are marked ‘-’. All continuous variables were scaled (mean = 0 ± 1 SD) prior to separating the breeding stages. Year and pair ID were fitted as random intercepts, and partner’s previous trip duration was fitted as a random slope in all models. Retained interaction terms and associated fixed effects were not averaged (marked ‘NA’) (see tables S5 & S6 for full coefficient report).

|  | Focal bird variables only | | | Partner bird variables only | | |
| --- | --- | --- | --- | --- | --- | --- |
|  | Retained in final model | Model averaged estimate | Standard error | Retained in final model | Model averaged estimate | Standard error |
| Effects on individual shift duration |  |  |  |  |  |  |
| Intercept | Y | 8.95 | 0.30 | Y | 8.71 | 0.30 |
| Focal age | N |  |  | - |  |  |
| Focal age^2^ | N |  |  | - |  |  |
| Partner age | - |  |  | N |  |  |
| Partner age^2^ | - |  |  | N |  |  |
| Focal boldness | Y | NA | NA | - |  |  |
| Partner boldness | - |  |  | Y | -0.07 | 0.10 |
| Date | Y | -1.26 | 0.42 | Y | -1.24 | 0.42 |
| New partner ­–true | Y | 0.33 | 0.37 | Y | 0.31 | 0.38 |
| Partner’s previous trip duration | Y | 0.15 | 0.16 | Y | 0.16 | 0.16 |
| Sex ­–male | Y | NA | NA | Y | -0.08 | 0.15 |
| Interactions acting on individual shift duration |  |  |  |  |  |  |
| Focal boldness x focal age | N |  |  | - |  |  |
| Focal boldness x focal age^2^ | N |  |  | - |  |  |
| Focal age x partner’s previous trip duration | N |  |  | - |  |  |
| Focal age^2^ x partner’s previous trip duration | N |  |  | - |  |  |
| Focal boldness x partner’s previous trip duration | N |  |  | - |  |  |
| New partner-true x partner’s previous trip duration | N |  |  | N |  |  |
| Partner boldness x partner age | - |  |  | N |  |  |
| Partner boldness x partner age^2^ | - |  |  | N |  |  |
| Partner age x partner’s previous trip duration | - |  |  | N |  |  |
| Partner age^2^ x partner’s previous trip duration | - |  |  | N |  |  |
| Partner boldness x partner’s previous trip duration | - |  |  | N |  |  |
| Focal boldness x sex ­–male | Y | NA | NA | - |  |  |
| Partner boldness x sex ­–male | - |  |  | N |  |  |
| Age x sex ­–male | N |  |  | - |  |  |
| Age2 x sex ­–male | N |  |  | - |  |  |
| Partner age x sex ­–male | - |  |  | N |  |  |
| Partner age^2^ x sex ­–male | - |  |  | N |  |  |

Appendix S4: full report of top model sets

We report the coefficients of the top model set (the best fitting models Δ Akaike’s information criterion _c_ < 2, with nested models excluded) prior to model averaging for both the incubation (Table S1) and brooding models (Table S2).

Table S1. Coefficients generated by the top model set (with nested models removed) derived from the incubation global model.

| Model No. | Intercept | Date | Partner boldness | Partner's previous trip duration | Sex ­–male | Partner age | Partner age^2^ |
| --- | --- | --- | --- | --- | --- | --- | --- |
| 841 | 14.72 | 2.48 | -0.52 | 0.30 | -1.21 |  |  |
| 825 | 14.52 | 2.34 |  | 0.31 | -1.48 | 4.65 | -4.20 |
| 585 | 15.06 | 2.77 | -0.53 |  | -1.21 |  |  |
| 777 | 14.84 | 2.43 |  | 0.30 | -1.60 |  |  |
| 569 | 14.85 | 2.63 |  |  | -1.50 | 4.53 | -4.09 |
| 521 | 15.17 | 2.72 |  |  | -1.61 |  |  |

Table S2. Coefficients generated by the top model set (with nested models removed) derived from the brooding global model.

| Model No. | Intercept | New partnership ­–true | Date | Partner boldness | Focal boldness | | Partner's previous trip duration | Sex –male | Focal boldness x sex ­–male |
| --- | --- | --- | --- | --- | --- | --- | --- | --- | --- |
|  |  |  |  |  | Female | Male |  |  |  |
| 4195277 | 8.81 | 0.57 | -1.19 | -0.24 | -0.52 | -0.13 | 0.24 | -0.34 | 0.39 |
| 4195273 | 8.85 |  | -1.14 | -0.23 | -0.52 | -0.13 | 0.24 | -0.34 | 0.39 |
| 4195021 | 8.89 | 0.58 | -1.33 | -0.24 | -0.52 | -0.13 |  | -0.34 | 0.39 |

Appendix S5: visualisation of pair data

The following graphs were produced in order to visualise the data obtained from individual pairs of albatrosses. These give insight into pair sample sizes and variation in intercept and slope of coordination strength within the population. Both graphs represent scaled versions of the variable partner’s previous trip duration (mean = 0 ± 1 SD) as the predictor variable and the square root of individual trip duration as the response variable. The slopes for these graphs were generated from a simplified model (see figure headings) in order to better visualise the relationship between these variables within the population.


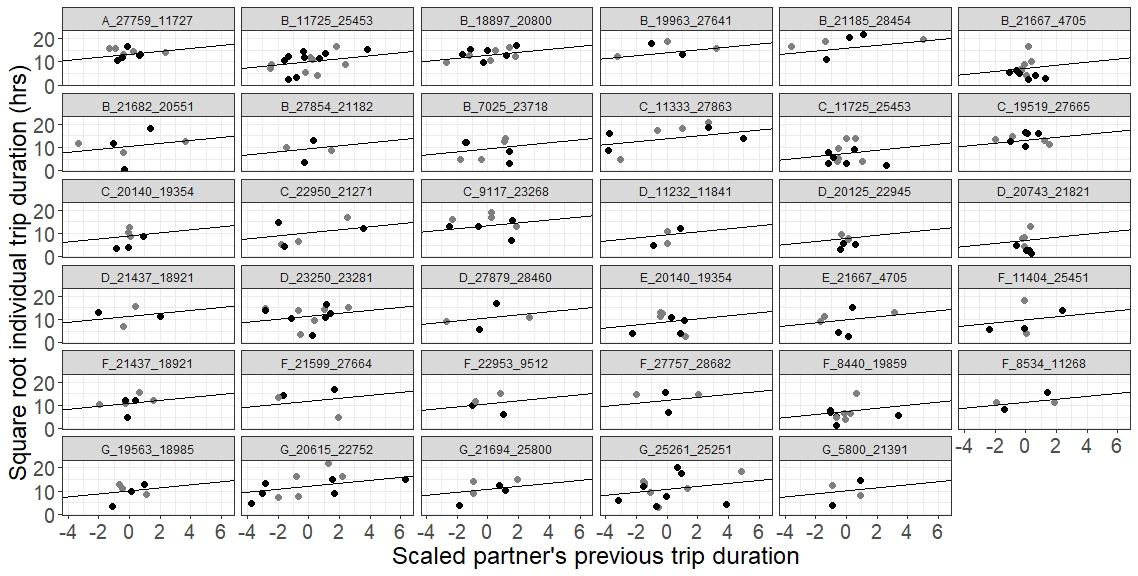


Fig. S1 Graphs representing the relationship between scaled partner previous trip duration (mean = 0 ± 1 SD) and square root individual trip duration for the breeding pairs of albatrosses included in the incubation model. In each panel, the female bird is shown in grey and male is shown in black. The slopes were generated using a simplified model (response variable: square root individual trip duration, predictor variable: scaled partner’s previous trip duration, random effects: scaled partner’s previous trip duration/cycle pair as a random intercept and slope) for more effective visualisation of the interpair differences. The data used to produce these models are anonymised. In each panel heading the letter represents the year of the breeding attempt and the two numbers represent the pair members’ IDs.


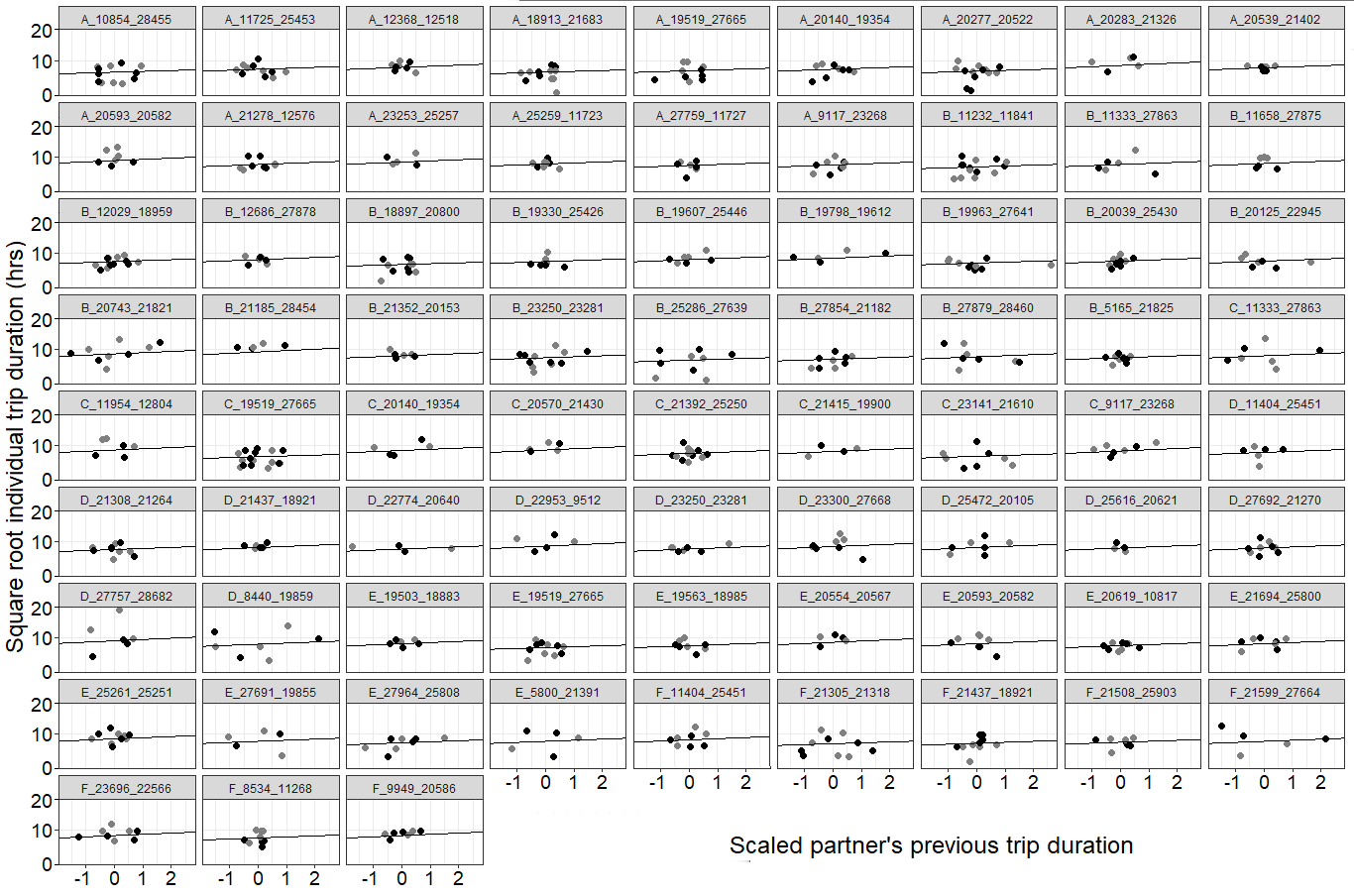


Fig. S2 Graphs representing the relationship between scaled partner previous trip duration (mean = 0 ± 1 SD) and square root individual trip duration for the breeding pairs of albatrosses included in the brooding model. In each panel, the female bird is shown in grey and male is shown in black. The slopes were generated using a simplified model (response variable: square root individual trip duration, predictor variable: scaled partner’s previous trip duration, random effects: scaled partner’s previous trip duration/cycle pair as a random intercept and slope) for more effective visualisation of the interpair differences. The data used to produce these models are anonymised. In each panel heading the letter represents the year of the breeding attempt and the two numbers represent the pair members’ IDs.
